# Internal replication of computational workflows in scientific research

**DOI:** 10.12688/gatesopenres.13108.2

**Published:** 2020-06-17

**Authors:** Jade Benjamin-Chung, John M. Colford, Jr., Andrew Mertens, Alan E. Hubbard, Benjamin F. Arnold

**Affiliations:** 1 Division of Epidemiology & Biostatistics, University of California, Berkeley, Berkeley, CA, 94720, USA; 2 Francis I. Proctor Foundation, University of California, San Francisco, San Francisco, CA, 94122, USA

**Keywords:** replication, reproducibility, masking, blinding, computational workflow

## Abstract

Failures to reproduce research findings across scientific disciplines from psychology to physics have garnered increasing attention in recent years. External replication of published findings by outside investigators has emerged as a method to detect errors and bias in the published literature. However, some studies influence policy and practice before external replication efforts can confirm or challenge the original contributions. Uncovering and resolving errors before publication would increase the efficiency of the scientific process by increasing the accuracy of published evidence. Here we summarize the rationale and best practices for internal replication, a process in which multiple independent data analysts replicate an analysis and correct errors prior to publication. We explain how internal replication should reduce errors and bias that arise during data analyses and argue that it will be most effective when coupled with pre-specified hypotheses and analysis plans and performed with data analysts masked to experimental group assignments. By improving the reproducibility of published evidence, internal replication should contribute to more rapid scientific advances.

## Introduction

A growing body of research has highlighted failures to replicate original study findings across disciplines ^1– 3^. Studies fail to be replicated for reasons ranging from unintentional coding errors to fraud ^4^. Even in the absence of errors or fraud, researchers’ own confirmation bias may impact the reproducibility of their findings ^5^. In response to mounting concerns, interest in methods to improve transparency and reproducibility in research has skyrocketed: researchers across disciplines have published recommended practices to improve reproducibility ^6– 11^ and detect lapses in publication integrity ^12^. In addition, certain funders have announced grant review criteria for rigor and reproducibility and dedicated funding for replication studies ^9, 13^.

A growing practice to diagnose reproducibility of the published literature is external independent replication, in which investigators outside an original study team attempt to replicate published results using the original dataset ^14^. More importantly, external replication may occur too late to prevent funding or policymaking based on erroneous results. For example, a landmark social science study was recently retracted due to a coding error ^15, 16^, yet the lead investigator had already received millions of dollars in funding based in large part on the initial study’s erroneous findings ^15, 17^. In addition, external replication may create an incentive for replicators to overturn original study findings that introduces bias and undermines constructive scientific discourse ^16, 17^. An alternative approach that does not create such an incentive is for journals to conduct “pre-publication review”, in which they attempt to replicate study findings using data and analytic code submitted prior to publication ^16, 17^. 

Here we describe “internal replication”, a process through which investigators from an original study team independently replicate a computational workflow in order to identify and resolve errors and help thwart biases that occur during computational analyses prior to publication ^10^. This practice is a natural complement to the growing practice of replicating experiments in different laboratories prior to publication in preclinical studies and has been recommended as a standard practice for computational workflows used by biologists ^18, 19^. In this article, we argue that adopting internal replication in scientific studies with a computational workflow should reduce errors and bias prior to publication. If adopted as a common practice, internal replication should improve the reliability of published evidence, increase the proportion of published findings that can be externally replicated, and increase the efficiency of the scientific process ^5^. Below we describe the workflow for internal replication and how the process can reduce errors and confirmation bias during computational analysis. 

### The status quo workflow

**Figure 1.**
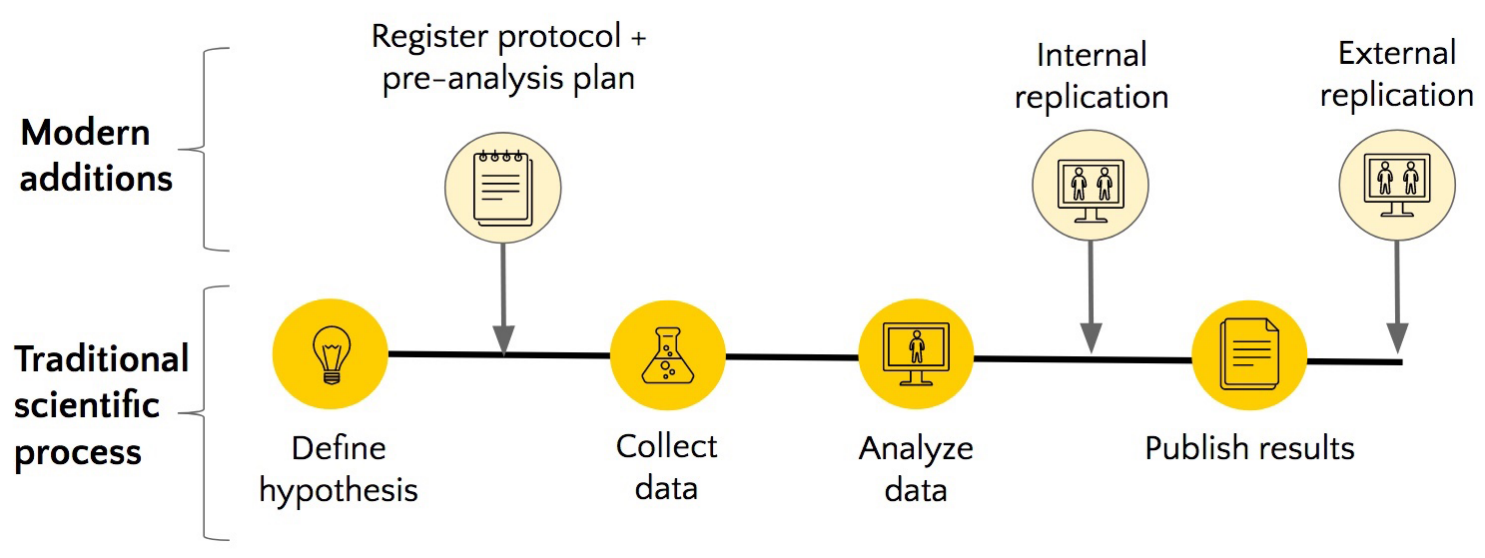
Modern additions to the traditional scientific process to increase rigor and reproducibility. Dark yellow circles indicate components of the traditional scientific process. Light yellow circles indicate modern additions to the scientific process.

In many disciplines, a typical computational workflow proceeds as follows: investigators conduct an experiment and/or collect data ( Figure 1). Afterwards, they often make decisions about computational analyses with full knowledge of experimental group assignment, and often a single analyst performs computation and error checking without independent replication prior to publication. Researchers naturally tend to confirm their own beliefs and are prone to making choices that – consciously or not – lead them to a statistically significant finding ^5^. In addition, it is common for researchers to thoroughly check unexpected results while errors in expected results may go unnoticed, introducing “disconfirmation bias” ^5^. Yet another threat to validity lies in human error. A typical computational workflow requires thousands of lines of code, and it is inevitable that some will include mistakes. Though only some mistakes will ultimately alter a study’s findings, occasionally a small error can amplify through an analysis like a genetic mutation, ultimately yielding vastly different results and policy implications. For example, a recent external replication ^20^ of a highly influential study that found externalities of school-based deworming identified a coding error in a variable used in a regression model; when corrected, one of the study’s most novel, policy-relevant findings (that worm infections were lower in control schools 3–6 km away from intervention schools) was closer to the null and no longer statistically significant ^21, 22^. These recent examples highlight the urgent need to improve computational analysis practices prior to publication to improve the accuracy of published literature. We argue that human error and confirmation bias are inevitable. Scientists need computational workflows that anticipate and minimize these cognitive bias traps.

Here, we discuss procedures for internal replication and explain how the practice can reduce unintentional errors and thwart bias during data analysis ^5^. Then we review internal replication practices we developed in eight internal replications we conducted for 32 outcomes in two large, cluster-randomized trials evaluating public health interventions in Bangladesh and Kenya ^23– 25^. 

## Methods

### The internal replication workflow

The internal replication workflow is a best practice that embraces confirmation bias and errors as an inevitable feature of scientific computation and reduces the likelihood that they will ultimately influence study findings. It complements other modern additions to traditional scientific practice, such as study registration and pre-analysis plans ( Figure 1). At a high level, this workflow consists of pre-specification of computational analyses before a study commences, masking of analysts to experimental group assignment, and internal replication of key results before publication.

***Including internal replication in pre-analysis plans***. Pre-specification of analysis plans prior to study commencement is an increasingly common best practice that should reduce confirmation bias. Pre-analysis plans define the study hypotheses and objectives, experimental groups or exposures, outcomes, and statistical analysis methods ^10^. Pre-analysis plans can also include plans for internal replication, including details about which analysis components will be replicated as well as the minimum allowable difference between independent analysts’ results (i.e., the tolerance level). For example, a tolerance level could be chosen so that any differences in results between replicators are small enough that they would not appear in published manuscripts. Pre-specifying internal replication procedures prior to study commencement minimizes the chance of confirmation bias during the replication process.

**Figure 2.**
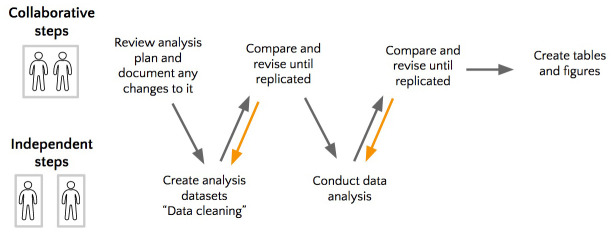
Internal Replication Workflow.

***Internal replication of key results before publication***. Following an experiment and/or data collection, the internal replication process begins when analysts independently prepare computational datasets by cleaning and merging raw data and generating variables ( Figure 2). During this step, they do not share their code with each other. Once each prepares an analysis dataset, analysts compare the values and summaries of each variable (e.g., the range and mean) between their datasets. If discrepancies exist, analysts work independently to resolve them and then iteratively compare and revise until their datasets are functionally identical (i.e., they have the same number of study units in each dataset and same values in each column). Once analysis datasets are replicated, the same process guides replication of computational analyses: analysts independently perform analyses, compare results, identify and resolve discrepancies between their results, and repeat the process until the difference in their results is less than the pre-determined tolerance level. Throughout the process, analysts may share their datasets and results with each other, but they do not share their code or analysis scripts until results are fully replicated. Code templates for comparing results while attempting to replicate are available from Zenodo ^26^, and programming tips for internal replication are available in Box 1. In studies with a high degree of repetition (e.g., multiple sites and outcomes) another tool to increase reproducibility is to create a software package using replicated code.

**Box 1. Programming tips for internal replication**

- **Decision log**: We recommend that independent data analysts keep a log of decisions they make together that are not covered by the pre-analysis plan. This includes how to handle unexpected outliers, discrepant identification numbers, and erroneous variable values. The log provides a thorough, transparent record of minor decisions made during the analysis.
- **Software**: Using statistical software such as R or Python that allows data analysts to efficiently load a large number of objects of differing dimensions (e.g., scalars, vectors, and matrices of different dimensions from different data sources), take the difference between them, and identify which are replicated facilitates replication. Other languages, such as Stata or SAS, allow objects of different dimensions to be loaded simultaneously, but the default is to work with a particular dataset with specific dimensions. As a result, while replicating, it may be more difficult to efficiently compare large numbers of matrices or other objects generated by each analyst to check for replication when using these languages. If analysts use the same software, we recommend that they use the same version of the software to ensure that differences in their results are not due to differences in software versions.
- **Version control**: We recommend using a version control system, such as Git, to track changes made during replication facilitates collaboration of data analysts during and after replication.
- **Variable type**: Agreeing upon the variable type, particularly for continuous variables, facilitates smooth replication. Some software truncates the number of significant figures when saving numeric variables in different formats. For example, since Stata stores numeric variables in binary, the value 0.1, which does not have a perfect binary representation, is stored differently for float and double variables types. These differences can carry forward and prevent replication.
- **Sorting and seed**: For analyses utilizing any kind of resampling (e.g. bootstrapping) or cross-validation, sorting and seed matter. Agreeing on variable sort order, sorting data at the same location in your script (e.g., right before analysis), and using the same seeds at the same locations facilitate replication.
- **Modular scripts**: Writing modular scripts that perform a limited number of discrete analyses makes it easier to diagnose failures to replicate. For analyses with thousands of objects, saving results in relatively small batches speeds replication by allowing data analysts to diagnose and resolve failures to replicate without having to re-run the entire analysis.
- **Bash scripts**: Bash scripts are plain text files that list a series of commands across different software packages; for instance, they could delete previous results objects and analysis logs from their stored location and then re-run analytic scripts in R or Stata. Separate bash scripts can be written for data management and different components of the analysis. Including code to remove all previously saved objects each time a script is re-run ensures that old versions of objects aren’t compared by accident when assessing whether replication was achieved.

**Figure 3.**
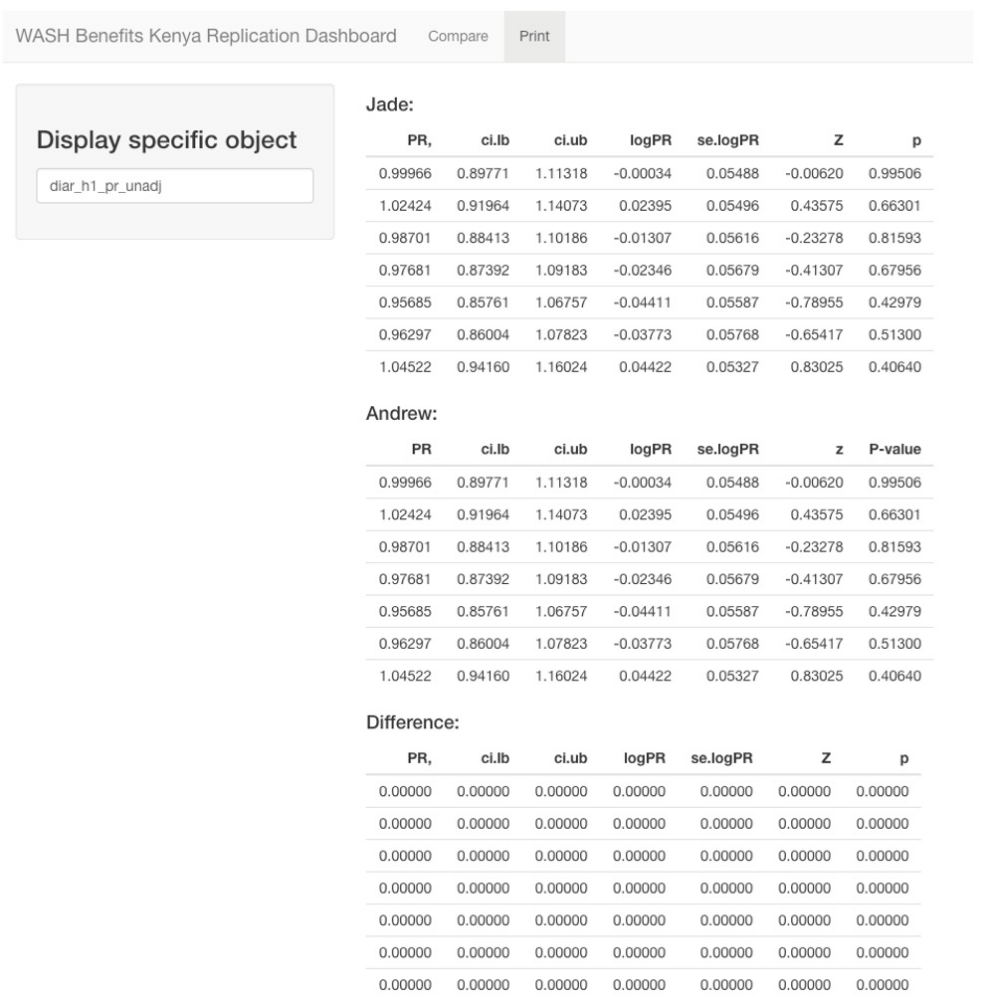
Screenshot of a Shiny R dashboard indicating that the diarrhea unadjusted prevalence ratios in WASH Benefits Kenya were replicated.

For example, the pre-analysis plan for the WASH Benefits Kenya trial included estimation of unadjusted prevalence ratios for diarrhea. Each analyst separately wrote the code in accordance with the pre-analysis plan using analysis datasets that they had ensured were functionally identical. Analyses were performed using *R* version 3.2.3. They saved estimates to a shared directory that could be accessed by both analysts ( link to analyst 1 code ^27^, link to analyst 2 code ^28^). We then compared each analyst’s estimates using a dashboard created with Shiny R to determine whether results were replicated. The dashboard displayed each analysts’ results, including the estimated prevalence ratio, confidence interval, log prevalence ratio, log of the standard error, Z-statistic, and p-value for the analysis in the columns, and each row displayed these estimates comparing each intervention arm to the control arm ( Figure 3). In addition, the dashboard showed the difference between each analyst’s estimates, which are all equal to 0, indicating that this analysis was internally replicated. We used this overall internal replication process for each component of the statistical analysis in this study (e.g., unadjusted analyses of other outcomes and adjusted analyses).

***Masked computational analyses***. Masking analysts to experimental group assignment, or in theory any other key variable during data preparation and analysis, can further reduce bias during internal replication. In a masked analysis, prior to working with data, an independent analyst re-randomizes the experimental group assignment variable. Subsequently, analysts use the re-randomized experimental group variable during dataset preparation and computational analysis. Results viewed during internal replication are scrambled, preventing any judgments that could produce favorable findings. Following internal replication, analysis scripts are re-run with the true experimental group assignment variable to obtain unmasked, final results. Though masked analyses are common in some fields, such as clinical trials ^29^ and particle and nuclear physics ^30^, to our knowledge the approach has not been widely adopted in other types of biomedical studies or in other fields, such as biology, psychology, or social sciences.

### Comparing internal replication to alternative approaches

***Internal replication vs. pair programming.*** At first glance, internal replication may resemble pair programming, a practice in which one analyst writes code while the other simultaneously reads and comments on the code to suggest coding strategies and improvements. Costs associated with pair programming and internal replication are likely to be similar since both approaches require two analysts to complete a single analysis. Unlike pair programming, in internal replication the vast majority of coding is done independently with minimal communication until data or results are compared. The advantage of this approach over pair programming is that it allows analysts to pursue completely different coding strategies which may be subject to differing sources of error and bias. Pair programming may be more subject to “group think” in which shared biases or judgment calls are reinforced or amplified. Thus, we believe that internal replication is more likely to identify failures to replicate results due to coding errors and biases than pair programming.

***Internal replication vs. pre-publication review***. One strategy to increase reproducibility that is complementary to internal replication is pre-publication review in which journals replicate study findings using replication scripts and datasets prior to accepting a manuscript for publication ^16, 17^. The *American Journal of Political Science* is one example of a journal that uses this approach. Internal replication before submission to peer review would catch errors internally before peer reviewers and journal editors consider a manuscript. Detecting errors after submission or after peer review is less efficient because it may require another round of peer review and revision. In addition, pre-publication review may be sufficient to ensure that the results generated by analytic code match those in the manuscript but may miss coding errors.

### How internal replication reduces bias

The act of comparing independently generated results during internal replication should reduce errors, confirmation bias, and disconfirmation bias. Analysts are unlikely to make identical mistakes, and discrepant results must be corrected in order to achieve replication. By requiring analysts to compare all of their results, internal replication improves the quality and extent of error checking, reducing disconfirmation bias.

The process of resolving discrepancies in independently generated results also reduces confirmation bias by illuminating judgment calls and decisions that may influence study results but are outside the scope of the pre-analysis plan. For example, decisions about how to handle erroneous responses to a coded survey question or missing responses for individual variables used to generate a composite variable cannot feasibly be included in pre-analysis plans. These types of small, seemingly inconsequential decisions often cannot be predicted before seeing the data. When differences in analysts’ decisions lead to discrepant results, internal replication provides a platform for transparent analytic choices outside the scope of pre-analysis plans.

Investigators must balance the need to reduce errors and bias with the significant costs required to perform internal replication. Internal replication can double the person-time required to complete an analysis and puts the burden of replication on the original study team. Yet, our view is that, overall, internal replication is far more efficient than external replication because the original study team is most knowledgeable about a study; external replication efforts require significant investment from the original investigator team because external replicators are not familiar with study materials ^16, 17^.

## Results

We developed best practices for internal replication while internally replicating data analyses for two randomized trials conducted in Bangladesh and Kenya named “WASH Benefits”. These trials measured the effect of single interventions (water (W), sanitation (S), handwashing (H), nutrition (N)) and combined interventions (combined W+S+H, combined W+S+H+N) on over 32 outcomes including child growth, diarrhea, parasite infection, and child development ^23– 25^. In addition, each trial tested 3 core hypotheses related to the effects of single interventions vs. combinations of interventions. The WASH Benefits trials were unusually complex and had a large number of interventions, hypotheses, and outcomes across two countries. Yet, it was exactly this complexity combined with the global importance of the results that motivated the study team to embrace internal replication. Our internal replication of these trials led us to uncover and resolve errors at every stage of the data analysis and brought to light numerous small judgment calls and assumptions made by each analyst. Correcting errors and transparently discussing data analysts’ assumptions helped us reduce bias in our study findings prior to publication. Trial data is available as underlying data ^31^.

### Including internal replication in pre-analysis plans

The WASH Benefits team published a protocol that described the study’s rationale, design, and analysis near the beginning of the study ^23^ ( Table 1). At the time of analysis but before working with the data, we updated the analysis plan for each country with additional details pertinent to specific analyses, and we registered the updated plans through the Open Science Framework (e.g., https://osf.io/63mna/). Having detailed pre-analysis plans in place improved the efficiency of internal replication by providing a clear roadmap for individual data analysts.

**Table 1.** Internal replication in the WASH Benefits trials.

Internal replication workflow steps	How each internal replication step reduces:			Examples from internal replication of WASH Benefits
	Confirmation bias	Disconfirmation bias	Human error	
1) Pre-specify computational analyses before study commences	Prevents p-hacking and analytic choices that produce favorable results by requiring investigators to make analytic decisions before seeing the data	Since all analyses – including secondary, subgroup, and sensitivity analyses – are pre- specified rather than selected post hoc, analysts incorporate them into the computational workflow and check them for errors in a systematic way	May indirectly reduce human error during analysis by reducing the number of decisions analysts must make during analysis, decreasing cognitive load	Prior to primary outcome data collection, the study investigators published a description of the study rationale, design, and analysis plan ^23^. After data collection but before analysis, investigators published minor modifications to the pre- analysis plan on the Open Science Framework ( https://osf.io/krezy).
2) Mask analysts to experimental group assignment	Prevents analysts from seeing study results during analysis	Results viewed during analysis are not meaningful because of re-randomized labels, so all results must be reviewed rather than only those that do not confirm expectations	May indirectly reduce human error during analysis by shifting attention from interpreting study findings to ensuring that the computational workflow is error-free	Prior to analysis, an independent analyst created a treatment variable that was randomly permuted within the trial’s randomized blocks. This scrambled treatment variable was used during data cleaning and analysis and was replaced by the real treatment assignments only after step 3 (below) was complete.
3) Internal replication of key results before publication	If there are any discrepancies in analytic decisions outside the scope of the pre- analysis plan between independent analysts, these are likely to prevent internal replication, requiring analysts to transparently discuss and agree upon major and minor analytic decisions	Requires every result to be compared and replicated, not only those that fail to confirm expectations	Catches and resolves numerous potential errors during data cleaning and analysis since such errors are likely to prevent internal replication	Investigators first internally replicated the analysis of primary outcomes at one site. Then using that internally replicated code, they developed a software package using internally replicated for use in analyses of secondary and tertiary outcomes that standardized output into a standard format ( https://github.com/ben- arnold/washb).

Caption: The WASH Benefits trials were two randomized, controlled, epidemiologic field trials conducted in Bangladesh and Kenya that measured the effect of single and combined interventions water, sanitation, handwashing, and nutrition interventions on over 32 outcomes including child growth, diarrhea, parasite infection, and child development ^23– 25^. Each trial tested 3 core hypotheses in 6 intervention arms related to the effects of single interventions vs. combinations of interventions. The complexity of the trials and anticipation of the trials’ results in the global health sector motivated the study team to perform internal replication.

### Masked computational analyses & internal replication of key results before publication

To reduce potential bias, analysts were masked to treatment assignments; we performed analyses using scrambled treatment assignment labels instead of real ones. During masked analyses, we encountered numerous differences in judgment calls that initially prevented replication. For example, two data analysts calculated age in months by dividing age in days using different numbers for average days per month. When age was used to calculate the height-for-age Z-score, the small differences in age in months had ripple effects that produced different results, particularly for effects that were borderline significant. We developed a workflow in which analysts kept notes about their judgement calls in a shared document, and they used these to reconcile differences and to make joint decisions in advance of future analyses to reduce replication time.

Another difference between analysts that initially prevented replication was the use of different software (Stata vs. R) and different data structures. For example, in an analysis of child growth data collected at two time points, one analyst happened to create a single dataset including both time points, and another included a separate dataset for each time point. The process of merging datasets with additional covariates against these two differing data structures created discrepancies in results that the replicators had to discuss and resolve. A concrete example of a mistake we caught that affected results was in the coding of the variable for the month of data collection. The variable was intended to be coded as a set of indicator variables when it was included in adjusted statistical models. One analyst accidentally coded it as a numeric variable, (e.g., 1 for January, 2 for February, etc.). The analysts were unable to replicate results until this discrepancy had been resolved. These examples illustrate the value of internal replication for detecting and resolving errors. 

After completing our first replication for WASH Benefits, we developed a R software package ^32^ for the trials for internal use based on the replicated code. The package streamlined the analysis across additional outcomes in the trials by providing a consistent template for analyses and standardizing output into a single, coherent format. Beyond the benefits of efficiency, the package’s consistent interface and internal data handling reduced the number of steps that each analysis needed to replicate. The time required to create the software package was justified since WASH Benefits was conducted in two countries and measured numerous outcomes. For studies with fewer iterations of the same type of analysis, the time investment required to develop a software package would likely outweigh its benefits. For any internal replication, creating a dashboard, such as the one we created with Shiny R ( https://osf.io/xbyrn/), to compare analysts’ estimates greatly increases the efficiency of internal replication by rapidly identifying estimates that failed to replicate. Advances in data science have made package and application development much easier, and we anticipate that in future years the process will become even more streamlined, making this approach feasible for studies with limited funding for internal replication.

## Conclusions

The internal replication tools we presented range from relatively easy (masking analysts to treatment assignment) to more resource intensive (developing an analytic software package). If resources are limited, replicating the analysis steps that are most error-prone and require the most judgment calls is a good place to start. For example, unglamorous data cleaning and processing steps are likely to be more error-prone since they require significantly more arbitrary decisions and complex programming steps than the computational analysis, especially when a pre-analysis plan is used. Alternatively, investigators could replicate a subset of outcomes or comparisons or a key portion of the analysis. In some disciplines research is conducted alone, and in this case a single analyst could partially replicate their own work by programming the analysis in alternative software packages (e.g., R vs. Stata) or by writing the same error-prone section of code twice.

Internal replication is one of many tools available to researchers to increase the reproducibility of their work, including publication of pre-analysis plans and publishing analytic datasets. A large proportion of studies are unable to publish their datasets due to human subjects protections or other privacy restrictions. In these cases, since external replication may not be possible, internal replication is even more valuable.

A limitation of internal replication is that it is performed by analysts from the same study team, who may make the same judgment calls or mistakes due to “group think”. Internal replication cannot detect identical errors or judgment calls made by each analyst. Nevertheless, our view is that internal replication can still prevent the majority of errors and biases, and publicly posting analysis plans and complete replication files allows external investigators to vet judgement calls.

Internal replication should increase the accuracy of published scientific results, thereby increasing the efficiency of the scientific process ^33^. Furthermore, it can reduce public controversies about how to interpret externally replicated results that differ from original results. These broader benefits should motivate funders to consider dedicated financial support for internal replication and spur journals to incentivize internal replication ^7, 16, 17^. For example, completion of internal replication could be a criterion editors use to assess studies during scientific journal peer review, and internally replicated studies could receive a reproducibility kite-mark or badge, such as those instituted by the journals *Psychological Science* and *Biostatistics* ^34– 36^ [Kidwell et al., Rowhani-Farid et al.]. Investigators who provide details of their plans for internal replication could be prioritized during grant proposal review. Including internal replication in the modern computational workflow allows scientists to embrace the fact that errors and bias are inevitable—a critical step towards advancing science and strengthening the culture of reproducibility.

## Data availability

### Underlying data

Open Science Framework: WASH Benefits Kenya Primary Analysis. https://doi.org/10.17605/OSF.IO/KREZY ^31^

This repository contains the following underlying data:

- washb-kenya-tr (This file contains the randomized treatment assignment for each cluster in the study. Available as in dta and csv format with codebook)
- washb-kenya-tracking (This file provides tracking information for the 8,246 households enrolled at the year 1 and year 2 visits. Available as in dta and csv format with codebook)
- washb-kenya-uptake-baseline (This file includes intervention adherence indicators collected at baseline in each household. Available as in dta and csv format with codebook)
- washb-kenya-uptake-midline (This file includes intervention adherence indicators collected at midline in each household. Available as in dta and csv format with codebook)
- washb-kenya-uptake-endline (This file includes intervention adherence indicators collected at endline in each household. Available as in dta and csv format with codebook)
- washb-kenya-midline-anthro (This file includes anthropometry measurements collected at midline in index children. Available as in dta and csv format with codebook)
- washb-kenya-endline-anthro (This file includes anthropometry measurements collected at endline in index children. Available as in dta and csv format with codebook)
- washb-kenya-diar (This file includes diarrhea illness symptoms collected at midline and endline in index children and children < 36 months in the compound. Available as in dta and csv format with codebook)

Data are available under the terms of the Creative Commons Attribution 4.0 International license (CC-BY 4.0).

## Software availability

Source code for the internal replication dashboard is available from: https://github.com/jadebc/replicate

Archived source code at the time of publication: https://doi.org/10.5281/zenodo.3626134 ^26^

License: Apache License 2.0

Source code for the WASH Benefits software package is available from: https://github.com/ben-arnold/washb

Archived source code at time of publication: https://doi.org/10.5281/zenodo.3626168 ^32^

License: GNU General Public License v3.0 or later

Source code for Analyst 1 of the WASH Benefits Kenya primary outcome analysis is available from: https://github.com/jadebc/WBK-primary-outcomes

Archived source code at the time of publication: https://doi.org/10.5281/zenodo.3627316 ^27^

License: Apache License 2.0

Source code for Analyst 2 of the WASH Benefits Kenya primary outcome analysis is available from: https://github.com/amertens/Wash-Benefits-Kenya

Archived source code at the time of publication: https://doi.org/10.5281/zenodo.3627359 ^28^

License: Apache License 2.0
