# Supplementary material for: Internal replication of computational workflows in scientific research
Source: Gates Open Res. 2020 Jun 17;4:17. Originally published 2020 Feb 5. [Version 2] doi: 10.12688/gatesopenres.13108.2 (PMC7403855; doi:10.12688/gatesopenres.13108.2)
Supplement: Peer review history

# Peer review: Reviewer response for version 2

**DOI:** 10.21956/gatesopenres.14341.r29139

**Published:** 2020-08-04

**Authors:** Lifeng Lin

**Affiliations:** 1 Department of Statistics, Florida State University, Tallahassee, Florida, USA

This manuscript illustrates the idea of internal replication for dealing with the crisis of research reproducibility and replicability in many fields. I like the framework and examples presented in this manuscript. I have the following specific comments:

  

- I think it might be helpful to use a few sentences to distinguish research reproducibility and research replicability in the introduction. It seems that this manuscript has used these two words in an exchangeable way, but I think the former may refer to the process that the research findings are exactly reproduced by re-performing the analyses, and the latter may refer to other studies conducted by other research teams (and thus based on different data) that produce consistent findings.
- In the second paragraph in the introduction section, the authors provided an example of coding error in a social science study. Similar issues also occurred in medical research. For example, in a recent clinical trial published in JAMA (Aboumatar *et al.*, 2018 ^1^), the treatment labels were erroneously switched in the analyses, leading to reversed findings, and the article was subsequently retracted (Aboumatar *et al.*, 2019 ^2^). It seems that such errors could be avoided by using the internal replication approach described by the authors.
- In the subsection of “Including internal replication in pre-analysis plans”, the authors mentioned that: “a tolerance level could be chosen so that any differences in results between replicators are small enough that they would not appear in published manuscripts.” I think the authors may further clarify how such differences may arise and how the tolerance level should be set. It seems that the authors referred to potential computational inaccuracies (e.g., due to rounding errors) or differences between methods used to implement statistical analyses. Although the authors provided an example of the WASH study (Figure 3), this example gave identical results by two analysts. I was wondering how researchers should address the discrepancies that substantially exceed the tolerance level. For example, multiple statistical software programs can be used to implement generalized linear mixed models, but different programs could lead to noticeable differences (Zhang *et al.*, 2011 ^3^).
- I like Figures 1 and 2. In Figure 2, I was wondering if the last step, “create tables and figures,” should be also conducted by both/multiple analysts independently or at least cross-validated by the analysts. Errors may occur when generating tables and figures (which could even occur during the typesetting process by publishers), while tables and figures are important content for readers to understand study findings.
- In the subsection of “Internal replication vs. pre-publication review,” it might be helpful to elaborate how the pre-publication review can be feasibly implemented. To my knowledge, few journals in the field of statistics and biostatistics actually replicate study findings using replication scripts and datasets prior to accepting a manuscript for publication. Many statistical projects involve extensive computations (e.g., simulation studies, very high-dimensional data analyses), and some require high-performance computers to perform the analyses. It is not very feasible to ask reviewers to run replication scripts, especially given that most review tasks are volunteer. For medical projects, patient-level data may be also needed for replicating findings, and they may not be shared without proper de-identification processes.
- In the results section, the authors discussed about masked computational analyses. “To reduce potential bias, analysts were masked to treatment assignments; we performed analyses using scrambled treatment assignment labels instead of real ones.” This is not new in clinical trials. Many trials are double blinded; treatment assignments are masked not only for analysts, but also for patients and healthcare providers.
- At the end of this manuscript, I think the authors may consider adding some discussion for time-sensitive research. This topic may be particularly important during the covid-19 pandemic. There have been thousands of studies on covid-19; some provide timely evidence, but some have been found to be misleading (e.g., Mehra *et al.*, 2020 ^4^). The internal replication is clearly able to reduce error and bias; however, it also requires much more amount of time and effort paid by research teams, and it may delay the publication of important time-sensitive evidence.

Is the rationale for developing the new method (or application) clearly explained?

Yes

Is the description of the method technically sound?

Yes

Are the conclusions about the method and its performance adequately supported by the findings presented in the article?

Yes

If any results are presented, are all the source data underlying the results available to ensure full reproducibility?

Yes

Are sufficient details provided to allow replication of the method development and its use by others?

Yes

Reviewer Expertise:

Statistical methods

I confirm that I have read this submission and believe that I have an appropriate level of expertise to confirm that it is of an acceptable scientific standard.

## References

[1] : Effect of a Program Combining Transitional Care and Long-term Self-management Support on Outcomes of Hospitalized Patients With Chronic Obstructive Pulmonary Disease. *JAMA*.2018;320(22) : 10.1001/jama.2018.17933 10.1001/jama.2018.17933 PMC658308330419103

[2] : Notice of Retraction. Aboumatar et al. Effect of a Program Combining Transitional Care and Long-term Self-management Support on Outcomes of Hospitalized Patients With Chronic Obstructive Pulmonary Disease: A Randomized Clinical Trial.JAMA . 2018;320(22):2335-2343. *JAMA*.2019;322(14) : 10.1001/jama.2019.11954 10.1001/jama.2019.11954 31593277

[3] : On fitting generalized linear mixed-effects models for binary responses using different statistical packages. *Stat Med*.2011;30(20) : 10.1002/sim.4265 2562-72 10.1002/sim.4265 21671252PMC3175267

[4] : RETRACTED: Hydroxychloroquine or chloroquine with or without a macrolide for treatment of COVID-19: a multinational registry analysis. *The Lancet*.2020; 10.1016/S0140-6736(20)31180-6 10.1016/S0140-6736(20)31180-6 PMC725529332450107

# Peer review: Reviewer response for version 2

**DOI:** 10.21956/gatesopenres.14341.r28939

**Published:** 2020-07-13

**Authors:** Garret Christensen

**Affiliations:** 1 U.S. Census Bureau, Washington, DC, USA

The authors did a good job addressing my initial concerns. At this point I have no remaining concerns and give it my full approval.

Is the rationale for developing the new method (or application) clearly explained?

Yes

Is the description of the method technically sound?

Partly

Are the conclusions about the method and its performance adequately supported by the findings presented in the article?

Partly

If any results are presented, are all the source data underlying the results available to ensure full reproducibility?

Yes

Are sufficient details provided to allow replication of the method development and its use by others?

Yes

Reviewer Expertise:

Reproducibility, research transparency, poverty programs, applied econometrics, labor economics

I confirm that I have read this submission and believe that I have an appropriate level of expertise to confirm that it is of an acceptable scientific standard.

# Peer review: Reviewer response for version 1

**DOI:** 10.21956/gatesopenres.14274.r28542

**Published:** 2020-02-19

**Authors:** Garret Christensen

**Affiliations:** 1 U.S. Census Bureau, Washington, DC, USA

This article summarizes the internal replication (pre-publication double coding of data cleaning and analysis) of the WASH Benefits study, and describes the process so other projects can emulate the methods widely. I find it to be mostly well-written and its argument sound. I have mostly minor comments and suggestions, which are below. I do have a few larger topics that might be addressed:

 A. How does your method compare to journal post-acceptance pre-publication review, as currently done in several political science journals?

 B. How does your method work for solo researchers?

 C. How does your method work in a world of proprietary or confidential data that cannot be publicly released? Something like 40% of articles in a top economics journal use data that can't be released, and the Federal Statistical Research Data Centers produce important research from data that can't be publicly released. (This is probably a point in your favor, since it's harder to do external replications of non-sharable data.)

 D. How does your method compare in costs and benefits to pair programming?

 Details in order of appearance:

1. Introduction, paragraph 2: I'm not sure citation 15 argues for external replication, at least not exclusively. Doesn't it make the case for quasi-internal, or at least pre-publication review (by the journal itself, after acceptance but before publication)? Political science journals such as AJPS do this now. It's just a "does your code actually produce your tables" and not as thorough as your method, but I think you should clarify this and possibly compare or contrast this method of journal pre-publication review to your method.
2. "a landmark study was recently retracted...": In which field/on what topic?
3. Status quo workflow paragraph: I think the use of citation 21 as an example needs to be more nuanced. Which "one of the study's most novel, policy-relevant findings"? I believe the original study authors would disagree with that claim. For example, from the Commentary response (which could also be cited) to cite 21: "The argument in Davey *et al*., that deworming impacts on school participation are not robust to different statistical approaches, is based on several analytical errors." Anyway, the point that an external replication led to disagreement or even controversy is certainly valid.
4. Methods including internal replication paragraph: "regardless of units" is a little unclear. I think I get the point about ultimate display in a journal, but often coefficients will be scaled. For example, population or income will be divided by 10K or 1m to avoid having to display a bunch of zeroes. 
5. Box 1, Software: Using non-object-oriented languages. What exactly do you mean by "makes it more difficult to load objects of different dimensions and compare them simultaneously"? An example could help.
6. Variable type: truncating the number of sig figs: I assume you are talking about character vs. numeric. Is this not just a machine precision/binary representation thing that all languages/computers do? Please clarify.
7. Bottom of page 4/just after Box 1: add a link here to the dashboard. It appears later, but this is the first mention.
8. Last paragraph before results: You have not discussed the large world of solo investigators. How do they fit into this scheme?
9. Results, making computational analysis...paragraph: "we kept notes about their judgement calls...": This seems close to pair programming. Software teaching organizations seem to advocate for this, and say it reduces errors. How would your method compare in terms of cost and reliability? Did you consider pair programming?
10. Conclusions: It seems kite-marks are very similar to badges, as adopted in psychology? (https://fivethirtyeight.com/features/even-psychologists-respond-to-meaningless-rewards/) Are there differences worth mentioning, or maybe just cite both?

*Any opinions and conclusions expressed herein are those of the authors and do not necessarily reflect the views of the U.S. Census Bureau.*

Is the rationale for developing the new method (or application) clearly explained?

Yes

Is the description of the method technically sound?

Partly

Are the conclusions about the method and its performance adequately supported by the findings presented in the article?

Partly

If any results are presented, are all the source data underlying the results available to ensure full reproducibility?

Yes

Are sufficient details provided to allow replication of the method development and its use by others?

Yes

Reviewer Expertise:

Reproducibility, research transparency, poverty programs, applied econometrics, labor economics

I confirm that I have read this submission and believe that I have an appropriate level of expertise to confirm that it is of an acceptable scientific standard, however I have significant reservations, as outlined above.

# Response

**Published:** 2020-06-11

**Authors:** Jade Benjamin-Chung

Thank you for these constructive comments. Below we describe how we have addressed them in the revised manuscript. We feel that the revisions in response to these comments have strengthened the manuscript. 

**A. How does your method compare to journal post-acceptance pre-publication review, as currently done in several political science journals?**

Response: Thank you for raising this question. We have added the following section that compares internal replication to pre-publication review in the Methods section.

*“Internal replication vs. pre-publication review*

*One strategy to increase reproducibility that is complementary to internal replication is pre-publication review in which journals replicate study findings using replication scripts and datasets prior to accepting a manuscript for publication [Gertler et al., 2018]. The American Journal of Political Science is one example of a journal that uses this approach. Internal replication before submission to peer review would catch errors internally before peer reviewers and journal editors consider a manuscript. Detecting errors after submission or after peer review is less efficient because it may require another round of peer review and revision.”*

Gertler P, Galiani S, Romero M (2018) How to make replication the norm. In: *Nature*. http://www.nature.com/articles/d41586-018-02108-9. Accessed 9 Mar 2018

**B. How does your method work for solo researchers?**

Response: We have added the following text in the first paragraph of the Conclusions:

*“In some disciplines research is conducted alone, and in this case a single analyst could partially replicate their own work by programming the analysis in alternative software packages (e.g., R vs. Stata) or by writing the same error-prone section of code twice.”*

**C. How does your method work in a world of proprietary or confidential data that cannot be publicly released? Something like 40% of articles in a top economics journal use data that can't be released, and the Federal Statistical Research Data Centers produce important research from data that can't be publicly released. (This is probably a point in your favor, since it's harder to do external replications of non-sharable data.)**

Response: The internal replication process is even more important for papers that cannot publish their data. We have added the following to the Conclusions:

*“* *Internal replication is one of many tools available to researchers to increase the reproducibility of their work, including publication of pre-analysis plans and publishing analytic datasets. A large proportion of studies are unable to publish their datasets due to human subjects protections or other privacy restrictions. In these cases, since external replication may not be possible, internal replication is even more valuable.”*

**D. How does your method compare in costs and benefits to pair programming?**

Response: We have added the following text to the Methods section:

*“Internal replication vs. pair programming*

*At first glance, internal replication may resemble pair programming, a practice in which one analyst writes code while the other simultaneously reads and comments on the code to suggest coding strategies and improvements. Costs associated with pair programming and internal replication are likely to be similar since both approaches require two analysts to complete a single analysis.* *Unlike pair programming, in internal replication the vast majority of coding is done independently with minimal communication until data or results are compared. The advantage of this approach over pair programming is that it allows analysts to pursue completely different coding strategies which may be subject to differing sources of error and bias. Pair programming may be more subject to “group think” in which shared biases or judgment calls are reinforced or amplified. Thus, we believe that internal replication is more likely to identify failures to replicate results due to coding errors and biases than pair programming.”*

**Details in order of appearance:**

**1. Introduction, paragraph 2: I'm not sure citation 15 argues for external replication, at least not exclusively. Doesn't it make the case for quasi-internal, or at least pre-publication review (by the journal itself, after acceptance but before publication)? Political science journals such as AJPS do this now. It's just a "does your code actually produce your tables" and not as thorough as your method, but I think you should clarify this and possibly compare or contrast this method of journal pre-publication review to your method.**

Response: We agree, and we have revised the sentences citing this paper in the Introduction as follows:

*“* *In addition, external replication may create an incentive for replicators to overturn original study findings that introduces bias and undermines constructive scientific discourse* *[Gertler et al., 2018]* *.* *An alternative approach that does not create such an incentive is for journals to conduct “pre-publication review” in which they attempt to replicate study findings using data and analytic code submitted prior to publication [Gertler et al., 2018]* *.”*

Gertler P, Galiani S, Romero M (2018) How to make replication the norm. In: *Nature*. http://www.nature.com/articles/d41586-018-02108-9. Accessed 9 Mar 2018

Regarding pre-publication review, please see our response to item A above.

**2. "a landmark study was recently retracted...": In which field/on what topic?**

Response: The authors of the original study were in both sociology and public health departments, so we have revised the text to refer to the study as a *“landmark social science study”.*

**3. Status quo workflow paragraph: I think the use of citation 21 as an example needs to be more nuanced. Which "one of the study's most novel, policy-relevant findings"? I believe the original study authors would disagree with that claim. For example, from the Commentary response (which could also be cited) to cite 21: "The argument in Davey *et al*., that deworming impacts on school participation are not robust to different statistical approaches, is based on several analytical errors." Anyway, the point that an external replication led to disagreement or even controversy is certainly valid.**

Response: We have added additional details about the specific result we referred to in that section as shown below. We were referring to the finding of lower worm infections in control schools 3-6 km away from intervention schools, not to school participation results. We believe that our sentence stating that “one of the study’s […] findings” reflects the fact that the “pure” replication effort identified errors that only changed some, not all, of the original study’s findings when corrected. We have also cited the Hicks et al. commentary responding to Aiken et al. We have not added the Davey et al. citation because it is not strictly a replication, but included re-analyses using different statistical approaches than the original paper.

*“For example, a recent external replication [Aiken et al., 2015] of a highly influential study that found externalities of school-based deworming identified a coding error in a variable used in a regression model; when corrected, one of the study’s most novel, policy-relevant findings (that worm infections were lower in control schools 3-6 km away from intervention schools) was closer to the null and no longer statistically significant [Miguel & Kremer, 2004; Hicks et al., 2015].”*

Aiken AM, Davey C, Hargreaves JR, Hayes RJ (2015) Re-analysis of health and educational impacts of a school-based deworming programme in western Kenya: a pure replication. *Int J Epidemiol* 44:1572–1580 . https://doi.org/10.1093/ije/dyv127

Miguel E, Kremer M (2004) Worms: Identifying Impacts on Education and Health in the Presence of Treatment Externalities. *Econometrica* 72:159–217 . https://doi.org/10.1111/j.1468-0262.2004.00481.x

Hicks JH, Kremer M, Miguel E (2015) Commentary: Deworming externalities and schooling impacts in Kenya: a comment on Aiken et al. (2015) and Davey et al. (2015 *). Int J Epidemiol* 44:1593–1596 . https://doi.org/10.1093/ije/dyv129

**4. Methods including internal replication paragraph: "regardless of units" is a little unclear. I think I get the point about ultimate display in a journal, but often coefficients will be scaled. For example, population or income will be divided by 10K or 1m to avoid having to display a bunch of zeroes.**

Response: We have revised this sentence as follows:

***“*** *For example, a tolerance level could be chosen so that any differences in results between replicators are small enough that they would not appear in published manuscripts.”*

**

**5. Box 1, Software: Using non-object-oriented languages. What exactly do you mean by "makes it more difficult to load objects of different dimensions and compare them simultaneously"? An example could help.**

Response: We have revised this part of Box 1 accordingly:

***“Software:*** *Using statistical software such as R or Python that allows data analysts to efficiently load a large number of objects of differing dimensions (e.g., scalars, vectors, and matrices of differing dimensions from different data sources), take the difference between them, and identify which are replicated facilitates replication. Other languages such as Stata or SAS, allow objects of different dimensions to be loaded simultaneously, but the default is to work with a particular dataset with specific dimensions. As a result, while replicating, it may be more difficult to efficiently compare large numbers of matrices or other objects generated by each analyst to check for replication when using these languages. If analysts use the same software, we recommend that they use the same version of the software to ensure that differences in their results are not due to differences in software versions.”*

****

**6. Variable type: truncating the number of sig figs: I assume you are talking about character vs. numeric. Is this not just a machine precision/binary representation thing that all languages/computers do? Please clarify.**

Response: This is a good point, and we have revised this part of Box 1 accordingly:

*“ **Variable type**: Agreeing upon the variable type, particularly for continuous variables, facilitates smooth replication. Some software truncates the number of significant figures when saving numeric variables in different formats. For example, since Stata stores numeric variables in binary, the value 0.1, which does not have a perfect binary representation, is stored differently for float and double variable types. These differences can carry forward and prevent replication.”*

**7. Bottom of page 4/just after Box 1: add a link here to the dashboard. It appears later, but this is the first mention.**

Response: We have done so.

**8. Last paragraph before results: You have not discussed the large world of solo investigators. How do they fit into this scheme?**

Response: We have added the following text in the first paragraph of the Conclusions:

*“In some disciplines research is conducted alone, and in this case a single analyst could partially replicate their own work by programming the analysis in alternative software packages (e.g., R vs. Stata) or by writing the same error-prone section of code twice.”*

**9. Results, making computational analysis...paragraph: "we kept notes about their judgement calls...": This seems close to pair programming. Software teaching organizations seem to advocate for this, and say it reduces errors. How would your method compare in terms of cost and reliability? Did you consider pair programming?**

Response: We have added the following paragraph comparing internal replication and pair programming in the Methods section.

*“Internal replication vs. pair programming*

*At first glance, internal replication may resemble pair programming, a practice in which one analyst writes code while the other simultaneously reads and comments on the code to suggest coding strategies and improvements. Costs associated with pair programming and internal replication are likely to be similar since both approaches require two analysts to complete a single analysis. Unlike pair programming, in internal replication the vast majority of coding is done independently with minimal communication until data or results are compared. The advantage of this approach over pair programming is that it allows analysts to pursue completely different coding strategies which may be subject to differing sources of error and bias. Pair programming may be more subject to “group think” in which shared biases or judgment calls are reinforced or amplified. Thus, we believe that internal replication is more likely to identify failures to replicate results due to coding errors and biases than pair programming. ”*

**10. Conclusions: It seems kite-marks are very similar to badges, as adopted in psychology? (https://fivethirtyeight.com/features/even-psychologists-respond-to-meaningless-rewards/) Are there differences worth mentioning, or maybe just cite both?**

Response: Yes, our understanding is that kite-marks and badges are essentially the same (see the Rowhani-Farid citation below). We have revised the text to use both terms and included new citations as well.

Kidwell MC, Lazarević LB, Baranski E, Hardwicke TE, Piechowski S, Falkenberg L-S, Kennett C, Slowik A, Sonnleitner C, Hess-Holden C, Errington TM, Fiedler S, Nosek BA (2016) Badges to Acknowledge Open Practices: A Simple, Low-Cost, Effective Method for Increasing Transparency. PLoS Biol 14:e1002456 . https://doi.org/10.1371/journal.pbio.1002456

Rowhani-Farid A, Barnett AG. Badges for sharing data and code at Biostatistics: an observational study. *F1000Res* 2018; **7**. DOI: 10.12688/f1000research.13477.2.
